# Identification of Persuasive Antiviral Natural Compounds for COVID-19 by Targeting Endoribonuclease NSP15: A Structural-Bioinformatics Approach

**DOI:** 10.3390/molecules25235657

**Published:** 2020-12-01

**Authors:** Mohd Saeed, Amir Saeed, Md Jahoor Alam, Mousa Alreshidi

**Affiliations:** 1 Department of Biology, College of Sciences, University of Ha’il, Ha’il 2440, Saudi Arabia; j.alam@uoh.edu.sa (M.J.A.); mo.alreshidi@uoh.edu.sa (M.A.); 2 Department of Clinical Laboratory Sciences, College of Applied Medical Sciences, University of Ha’il, Ha’il 2440, Saudi Arabia; am.saeed@uoh.edu.sa

**Keywords:** SARS-CoV-2, NSP15, virtual screening, molecular dynamics, natural compounds

## Abstract

SARS-CoV-2 is a positive-stranded RNA virus that bundles its genomic material as messenger-sense RNA in infectious virions and replicates these genomes through RNA intermediates. Several virus-encoded nonstructural proteins play a key role during the viral life cycle. Endoribonuclease NSP15 is vital for the replication and life cycle of the virus, and is thus considered a compelling druggable target. Here, we performed a combination of multiscoring virtual screening and molecular docking of a library of 1624 natural compounds (Nuclei of Bioassays, Ecophysiology and Biosynthesis of Natural Products (NuBBE) database) on the active sites of NSP15 (PDB:6VWW). After sequential high-throughput screening by LibDock and GOLD, docking optimization by CDOCKER, and final scoring by calculating binding energies, top-ranked compounds NuBBE-1970 and NuBBE-242 were further investigated via an indepth molecular-docking and molecular-dynamics simulation of 60 ns, which revealed that the binding of these two compounds with active site residues of NSP15 was sufficiently strong and stable. The findings strongly suggest that further optimization and clinical investigations of these potent compounds may lead to effective SARS-CoV-2 treatment.

## 1. Introduction

Viral diseases are a lethal threat to humans. New viruses emerge, and all major acute respiratory syndromes are affected [1]. SARS-CoV and MERS-CoV, among these, have high fatality rates. SARS-CoV-2 is now undergoing fast dissemination in a pandemic situation. The mortality rate of COVID-19 in the affected population is very high compared to those of SARS-CoV and MERS-CoV; it increased from 2% (January 2020) to more than 7% (May 2020), but is now globally decreasing (at present, 3.28%) [2]. Animal-to-human transmissions were observed in the past, but human-to-human transmissions were also recorded, and they formed the basis for the present pandemic [3].

SARS-CoV-2 is a positive-stranded RNA ((+) RNA) virus, one of a broad family of viruses that includes the Zika, chikungunya, and hepatitis C viruses. (+) RNA viruses bundle their genomes as messenger-sense RNA in infectious virions and only replicate these genomes in replication complexes by RNA intermediates [4]. Upon binding to the human host cell receptor (angiotensin-converting enzyme 2), the virus enters the host cell. The virus releases the RNA into the cytoplasm using host cell systems to begin the translation process; ORF1a and ORFab of the RNA are translated into polyprotein 1a and polyprotein ab, and are then processed by proteases and release nonstructural viral protein (NSP) [5,6].

The genomic content of SARS-CoV-2 is 70%–80%, such as that of SARS-CoV [7]. The NSP15 protein from SARS-CoV-2 is 89% identical to the SARS-CoV protein. Studies showed that inhibition of NSP15 can slow down viral replication. This suggests that drugs designed to target NSP15 could be developed as effective anti-COVID-19 drugs. NSP15 is also referred to as a uridylate-specific endoribonuclease, as it ideally cleaves the 3′ end of uridines. It is believed to perform a crucial task in viral replication in host cells [8].

Natural drug treatments can help to manage infection transmission. Nature offers a large array of chemicals yet to be discovered to produce medicines for the treatment of various viral diseases. Analysis to identify natural drugs against SARS-CoV-2 targets is still underway. Few natural products were shown to have antiviral activity at nanomolar concentrations that lead to further drug growth [9]. Computational study offers an evaluation of the future consequences of the discovery of lead molecules targeting SARS-CoV-2-interactive proteins in infected patients [10]. Following previous computational attempts to identify possible lead/drug molecules against SARS-CoV-2 NSP15, a virtual screening of the natural compounds library (Nuclei of Bioassays, Ecophysiology and Biosynthesis of Natural Products (NuBBE) database) against the active site of the NSP15 was conducted in this study.

The present study included multistep virtual screening and indepth molecular docking followed by a molecular-dynamics (MD) simulation report of the two best-scoring ligands that exhibited better affinity with the target protein. These compounds may further be tested against COVID-19 in the forthcoming days.

## 2. Results

### 2.1. Virtual Screening

**Figure 1:**
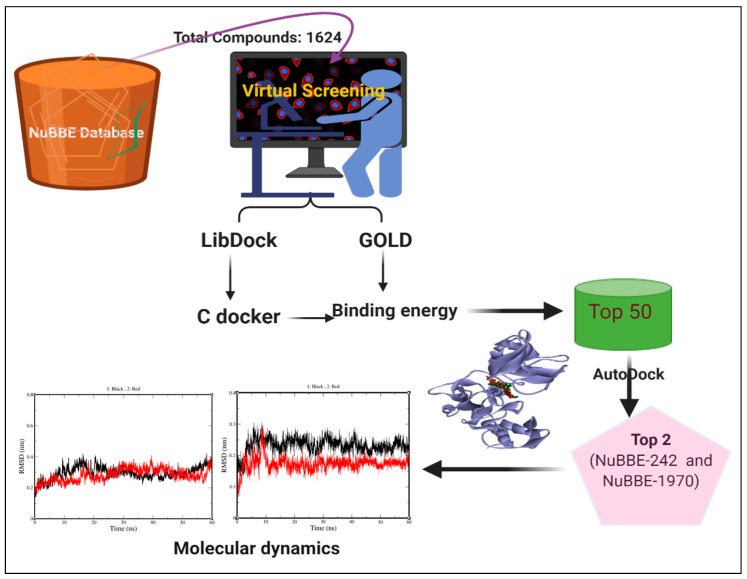
Flow diagram of virtual-screening steps.

This study aimed to identify effective phytochemical compounds that could actively hinder the activity of the SARS-CoV-2 virus. Here, we selected an important target protein from the SARS-CoV-2 genome, NSP15 [11,12]. The NSP15 protein was chosen for ligand docking, which was also confirmed by different studies in the literature as the most druggable site. With the high failure rates, high costs, and sluggish speed of modern drug research and production, the reuse of molecules and medications for the treatment of diseases is the easiest way to identify potential leads [13]. From the multistep route of virtual screening of the prepared NuBBE library, we filtered out the 10 best hits by successive screening; the two top-scoring hits (higher binding energy), NuBBE-242 and NuBBE-1970, were selected for detailed interactions studies and MD simulation (Figure 1).

Indepth molecular-docking analysis of screened compounds NuBBE-242 and NuBBE-1970 with the active site of NSP15 was performed to simulate and visualize protein–ligand interactions. NSP15 was found to interact with NuBBE-1970 through 11 amino acids (aa), namely, GLY165, VAL166, THR167, LEU168, ILE169, ARG199, ASN200, GLU203, PHE204, LYS205, and ARG207. NuBBE-242 interacted with 13 aa: LYS90, GLY165, VAL166, THR167, LEU168, ILE169, GLN197, SER198, ARG199, ASN200, LYS205, PRO206, and ARG207. Of these residues, three different aa, THR167, ASN200, and ARG207, were involved in H-bond interaction for the NuBBE-1970–NSP15 complex, and four different aa were involved in hydrophobic interaction (VAL166, ILE169, ARG199, LYS205).

Three H bonds and four hydrophobic interactions were observed in the complex stabilization of NuBBE-242–NSP15. PRO206, GLY165, and ARG207 were found in H-bond interactions, and four different aa, ARG199, LYS205, THR167, and VAL166, were found in hydrophobic interactions.

**Figure 2:**
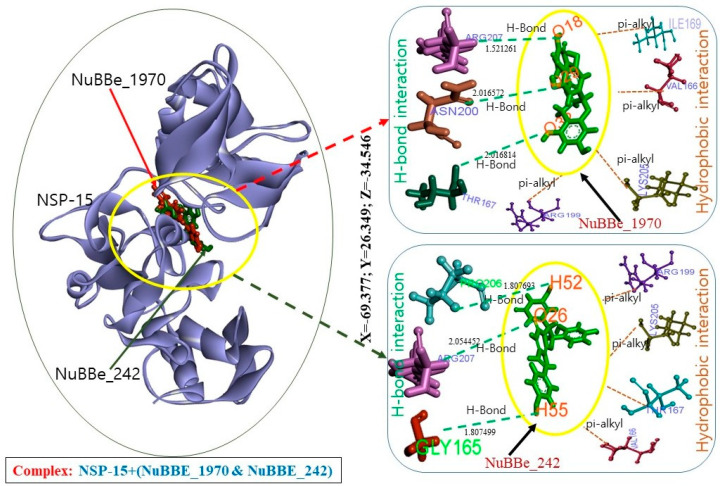
Complex structures of NuBBE-1970 and NuBBE-242 with active site of NSP15.

NuBBE-1970 and NuBBE-242 were found to interact at the NSP15 active site (Figure 2), the coordinates of which were X = −69.377, Y = 26.349, Z = −34.546. At this active site, GLY165, VAL166, THR167, LEU168, ILE169, ARG199, ASN200, LYS205, PRO206, and ARG207 were found in common interactions, which was also supported by published articles [14,15]. Table 1 displays all of these interacting aa in various bindings.

The binding energies of complexes NuBBE-1970–NSP15 and NuBBE-242–NSP15 were found to be −483.6 and −305.872 kcal/mol, respectively, which were quite high among the selected compounds. The GOLD fitness scores of these two compounds were found to be 72.2588 and 78.5397, respectively. Besides this, further docking analysis by Autodock [16] was performed to check the inhibition of the selected compounds along with different parameters, namely, their binding energy, inhibition constant, intermolecular energy, and electrostatic energy, as shown in Table 2.

### 2.2. Pharmacokinetic Properties

To explore the behavior of the selected compounds within an organism, their absorption, distribution, metabolism, and excretion (ADME) were predicted by SwissADME [17] and pkCSM tools. Several therapeutic compounds failed to reach clinical trials because of unfavorable ADME parameters. Lipinski’s rule of five [18], Veber’s rule [19], Egan’s rule [20], polar surface area (TPSA), and a number of rotatable bonds were predicted and are explained in Table 3.

### 2.3. Molecular-Dynamics Simulation

**Figure 3:**
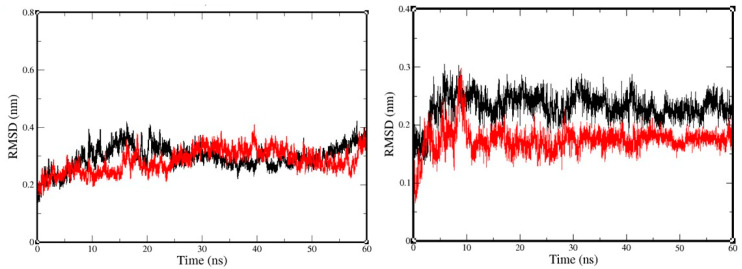
Root-mean-square deviations (RMSDs) of backbone atoms of each complex and of ligands (right plot). Black, NuBBE-1970–NSP15 complex; red plot, NuBBE-242–NSP15.

MD simulation was performed for 60 ns for both complexes. The constancy of the trajectories was examined via root-mean-square deviation (RMSD). Figure 3 reports the RMSD of the backbone atoms for the receptor and ligand separately for selected complexes NuBBE-1970–NSP15 and NuBBE-242–NSP15. For backbone atoms, it was found that RMSD changes in all systems were initiated at the same point, ~0.125 nm. The black line represents the RMSD value change in the backbone of NuBBE-1970–NSP15 that indicates few oscillations until ~30 ns; after this time, RMSDs were observed to be steady and remained so until the end of the simulation, with an average value of around 0.37 nm.

RMSD values in the NuBBE-242–NSP15 backbone (Figure 3, red plot) showed turbulence from ~26 ns that remained until ~57 ns; however, a little oscillation was observed for ~2 ns at the end of the simulation. Considering the difference between RMSDs of 1 nm from ~40 ns to the end of the simulation, the RMSD of the backbone atoms in both complexes appeared to be stable.

The RMSD plot of the ligands within both complexes (Figure 3, right plot) showed a sudden elevation at a time within the first 10 ns, and the maximal RMSD value was noted as 0.3 nm. However, RMSD values for the ligand in NuBBE-1970–NSP15 (black) formed a plateau after ~10 ns, and the average value was 0.26 nm; the ligand in NuBBE-242–NSP15 was found to be steady after ~18 ns. Consequently, the RMSDs of the ligands of the two above complexes were found to have constant trajectories during the simulation, unlike those of the backbone atoms, indicating that the ligands showed high stability within the close contact residues of the protein receptor.

Flexible residues of both complexes (NuBBE-1970–NSP15 and NuBBE-242–NSP15) were analyzed through root-mean-square-fluctuation (RMSF) plots (Figure 3—black, NuBBE-1970–NSP15; red, NuBBE-242–NSP15) in compliance with X-ray data. RMSF values were recorded when the most obvious flexible residues were recruited, which were found to be comparatively higher in NuBBE-1970–NSP15 than those in NuBBE-242–NSP15.

**Figure 4:**
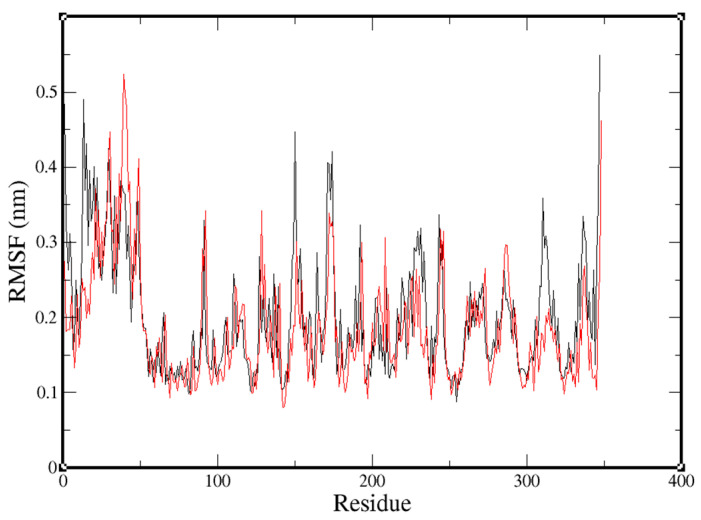
Representation of root mean square fluctuation (RMSF) of each complex. Black, NuBBE-1970–NSP15 complex fluctuations; red, NuBBE-242–NSP15 complex fluctuations.

For the stability of the complex, RMSD was determined, and found to become steady and remain so until the end of the simulation; the average value was around 0.37 nm. Ignoring residues of the N- and C-terminals, the maximal RMSF value for all simulations reported here (~0.35 nm) was detected in neighboring residues of the active site. For NuBBE-1970–NSP15 and NuBBE-242–NSP15, the range in residues 135–175 was employed in the width of the high RMSF peak. The corresponding pattern was found to be in good agreement with the secondary structure and with the B-factors reported for the initial structure (Figure 4).

**Figure 5:**
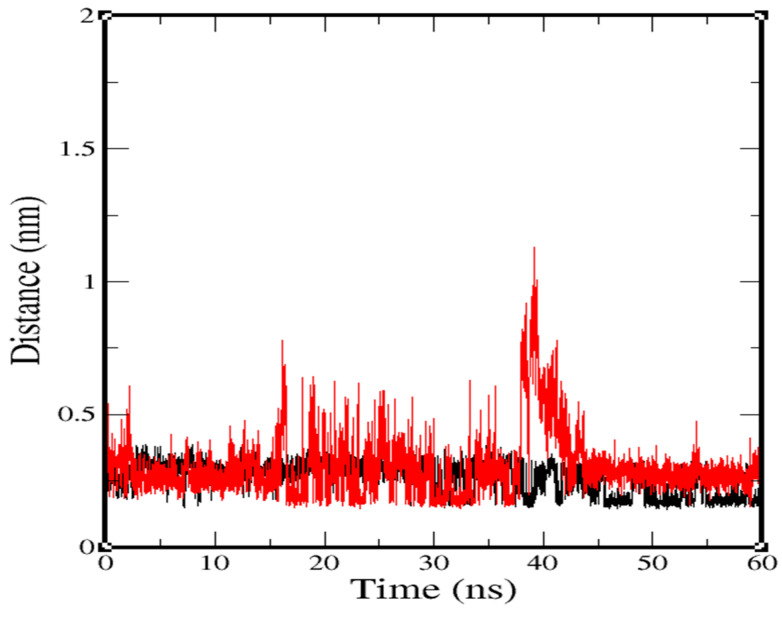
Calculation of minimal distances from ligand within 0.4 nm to close residues. Black, NuBBE-1970–NSP15; red, NuBBE-242–NSP15.

The minimal distances between residues of the protein receptor and both ligands were calculated, and found to have average values of 0.191 nm for NuBBE-1970–NSP15 and 0.26 nm for NuBBE-242–NSP15 (Figure 5). Fascinatingly, in the NuBBE-242–NSP15 complex from 38 to 43 ns (for 5 ns), the ligand was moved from the site and found more than 1 nm from the binding site. Although it was for a very short while, the ligand came back to site and remained stable till end of the simulation. This observation suggests that complex-1, i.e., NuBBE-1970–NSP15 was stabler than complex-2, i.e., NuBBE-242–NSP15.

**Figure 6:**
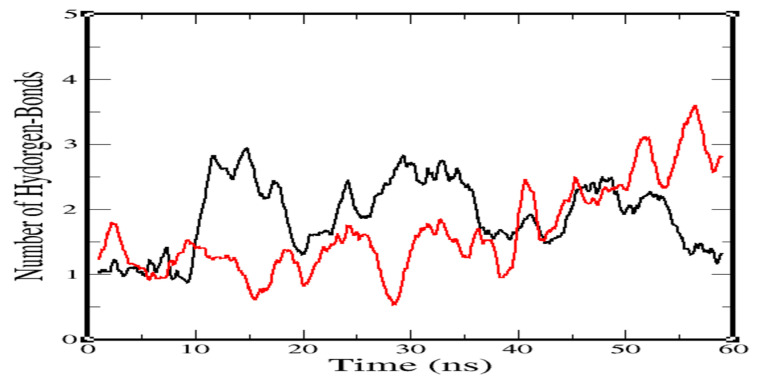
Representation of H-bond calculation during simulation, signifying observation. Default cut-off of 0.35 nm was considered.

Besides this, the robustness of ligands was measured by H-bond approximation at the cavity (Figure 6) using a cut-off of 0.35 nm. On average, two H bonds were formed between the cavity residues of NuBBE-1970–NSP15 and the atoms of the ligand during the simulation. Two H bonds were found in NuBBE-1970–NSP15 for most of the simulation time, around ~50 ns. Between ~10 and 50 ns of the simulation, two H bonds were considered constant; afterward, fair fluctuation occurred. In NuBBE-242–NSP15, an average of three H bonds was obtained, demonstrating more robust interaction between cavity residues and ligand. During the simulation, the number of bonds exponentially improved after ~30 ns and remained consistent until the end of the simulation time.

**Figure 7:**
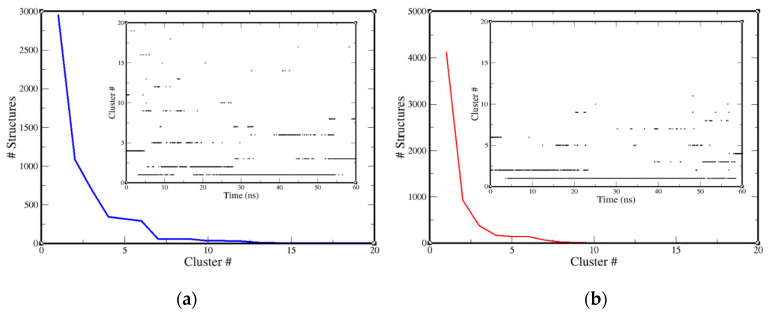
Cluster analysis of (**a**) NuBBE-1970–NSP15 and (**b**) NuBBE-242–NSP15 complexes by taking backbone for calculations. Descending blue and red lines indicate clusters of representation structures with respect to time. Subplots represent generated clusters during simulation. Cluster analysis was approximated using a cut-off of 0.15 nm.

The trajectories of both systems were subjected to cluster analysis (cut-off value, 0.15 nm) for the temporal distribution to secure convergence. Cluster distribution was categorized by a sequence of flat bars, each continuing for a longer time, suggesting a steady evolution towards a stable relaxed state of the complex. Cluster distribution patterns (Figure 7) were determined by using the trajectories of both systems in fast thermal equilibrium.

However, infrequently, the motion of the interior atom determined the creation of one less probable cluster. In Figure 7, a total of six slopes were found, and interestingly, the first slope of structure decline in the NuBBE-1970–NSP15 case was perceived from ~2900 to ~1100 structures; this observation possesses strong agreement with the subplot of cluster distribution with simulation time. In the end, an average of 10 clusters were found, where ~100 structures of similar confirmations were spotted. In the case of NuBBE-242–NSP15, a total of five slopes were marked. The first slope was observed from ~4100 to ~900 structures, where these structures were reserved in fewer than three clusters. At the end of the simulation, approximately 50 structures were observed, which were held in reserve for ~7 clusters.

The leading clusters in Figure 7 were in rapid equilibrium, with almost full-lifetime structures that lasted for 60 ns; this implied that the system was in fast equilibrium, sampling many conformations that did not differ in their stability. For NuBBE-1970–NSP15, the backbone of the protein receptor was noisily executed, whereas the trajectory of NuBBE-242–NSP15 was less disorderly, and it was a plane of continuous change to a new conformation, signified by a series of clusters each having a long lifetime.

**Figure 8:**
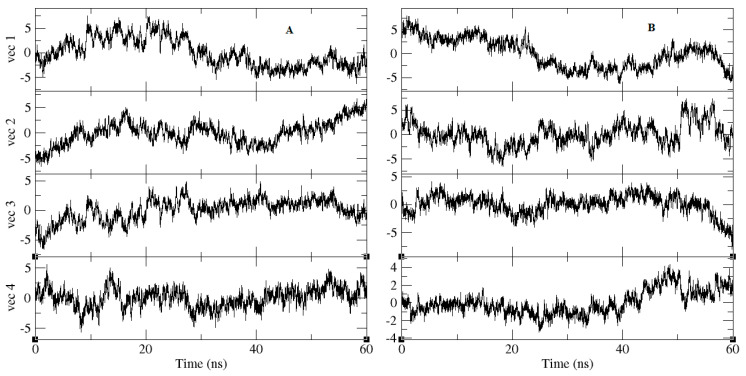
Principal-component analysis of trajectories of both complexes (**A**) NuBBE-1970–NSP15 and (**B**) NuBBE-242–NSP15. Projections of all four eigenvectors during simulation. Calculation was considered for total of 60 ns of simulation.

**Figure 9:**
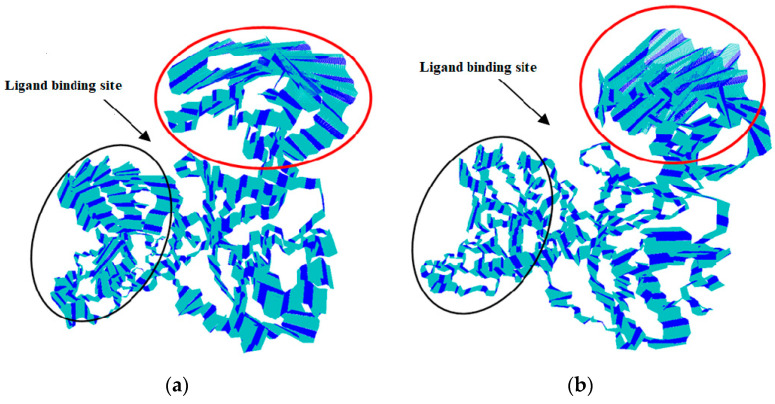
Movement of backbone atoms during simulation by 100 collectively aligned frames. (**b**) NuBBE-1970–NSP15 complex; (**a**) NuBBE-242–NSP15. Motion of backbone atoms of NuBBE-1970–NSP15 and NuBBE-242–NSP15 is as calculated by principal-component analysis (PCA). Comparison of the wide and narrow sections shown by red and black ellipses, where red ellipses showed tendency towards active ligand site (arrow). Width of bands was proportional to amplitude, signifying that atoms moved in a similar direction.

Principal-component analysis of the relaxation (calculated for backbone atoms) is presented in Figure 8 and Figure 9. The projection of eigenvectors is depicted in Figure 8. In this presentation, the molecule was allowed to move along the largest vector, corresponding with the dominant conformation transitions during the relaxation of the structure. Figure 9 describes the collection of frames of the protein backbone, where the width of the bands was proportional to the amplitudes the atom makes; narrow bands represent sections that hardly moved, while wide bands are the regions most affected by the transitions. As seen in Figure 9, the red circle, which was supposed to move towards the cavity where ligands had interacted, signifies that, in NuBBE-1970–NSP15, the region occupying the red ellipse showed a transition (wide band) towards the ligand within the cavity. On the other hand, the red-ellipse region demonstrated a significant move towards the cavity in NuBBE-242–NSP15, in strong agreement with the H-bond calculation. However, the black ellipses revealed wide- and narrowband regions for NuBBE-1970–NSP15 and NuBBE-242–NSP15, respectively, suggesting that the width of the band illustrated high amplitude, executing significant functionality.

In the case of NuBBE-1970–NSP15 backbone transitions, atoms attempted to allocate within 0.4 nm of the ligand-binding site, signifying greater functionality and stability with the ligand. Interestingly, one red and one black ellipse of either complex showed stability and interaction with ligands during the simulation. A video of the principal components of both complexes can be found in the Supplementary Material. Considering the area space between the black and red ellipses in both complexes indicated a noteworthy role in finding close contact residues within 0.4 nm of the ligand atoms. Therefore, NuBBE-1970–NSP15 showed greater robustness and functionality with the ligand, although during the initial stage of simulation, more conformational changes were examined until the ligand in the binding site found stability.

## 3. Discussion

With the rising global number of SARS-CoV-2 infection cases, it is imperative to discover potential therapies in order to cure this devastating disease. Due to their rarity, phytocompounds are important, but due to their weak pharmacokinetics, only few reach the production stage [21]. Recently, chloroquine and hydroxychloroquine were found to have promising in vitro and clinical results [22], but these drugs are highly toxic, and disrupt heart and neurological functions [23,24]. To address the current problems, we utilized a novel approach by collecting natural compounds to check their potential inhibitory action against the critical SARS-CoV-2 target.

The screening was performed using the Discovery Studio 2020 (DS 2020) suite with LibDock as a high-throughput screening tool, followed by docking optimization by CDOCKER and final scoring by calculating binding energies. The prepared library was also screened by GOLD docking software. NuBBE-1970 and NuBBE-242, observed as the common top-scoring compounds (from GOLD and DS screening), were selected and further studied in detail for the inhibition of the NSP15 target protein of COVID-19. The different scores for the ligand, protein, and its complex are shown in Table 4.

For the stabilization of complex NuBBE-1970–NSP15, H bonds, namely, THR167:HN–UNK0:O32, ASN200:HN–UNK0:O29, and ARG207:HE-UNK0:O18, with H-bond distances 2.016814, 2.016572, and 1.521261 Å, respectively, and hydrophobic interactions VAL166–UNK0, UNK0–ILE169, UNK0–ARG199, and UNK0–LYS205 play an important role. Along with these interactions, one electrostatic interaction was found with GLU203 through GLU203:OE1–UNK0.

UNK0:H52–PRO206:O, UNK0:H55–GLY165:O, and ARG207:HE–UNK0:O26 with H-bond distances 1.807693, 1.807499, and 2.054452 Å, respectively, with the ligand were found in the complex formation of NuBBE-242–NSP15. Hydrophobic interaction was also seen, namely, ARG199:HD1–UNK0:O26, LYS205:HE2–UNK0:O25, THR167:HN–UNK0, and UNK0–VAL166. It was reported that formed H bonds among ligands and receptors contribute to the stability of complexes [25,26,27].

Further, the pharmacokinetic properties of NuBBE-1970 and NuBBE-242 were calculated using SwissADME showing different parameters, for example, pharmacokinetics, drug likeness (Lipinski’s rule, Ghose’s rule, Egan’s rule (defines absorption of the drug molecule) and Veber’s rule (for oral bioavailability)), lipophilicity, water solubility, and physicochemical properties [19,20,28]. NuBBE-1970 showed one violation, while NuBBE-242 showed two violations in Lipinski’s rule estimation. However, log S solubility values of the tested ligands were between −4.06 and 0.25 (Table 3). Water solubility is an important constraint for any drug molecule intended for administration via the oral route in sufficient amounts [29,30]. Water solubility is defined via two different parameters: solubility-Esol [31] and Ali [32] models. Log S (ESOL) values for NuBBE-1970 and NuBBE-242 were found to be −6.18 and −3.56, respectively. Log S (Ali) values were calculated as −7.53 and −4.91.

Considering binding-site residues within protein cavities, we recruited residues for further calculation; those were performed using these residues against the corresponding ligand. From a cut-off distance of 0.4 nm within the protein, we recruited close contact residues for NuBBE-1970–NSP15 and NuBBE-242–NSP15; these were GLU203, ASN200, LYS205, SER198, and ILE169. Remarkably, in the case of NuBBE-1970–NSP15, some atoms of the ILE169, THR167, ARG91, and LYS90 residues showed greater fluctuations from the ligand atom; consequently, the minimal distance plot demonstrated moderate inflation at the time of approximately 15 to 42 ns, but later (~17 ns) recovered a suitable distance of less than 0.25 nm. This observation promoted significant additional robustness and an equivalent binding phenomenon with ligands in both cases of the complex. However, ligands recovered the distance up to less than 0.25 nm in the last 20 ns and remained consistent until the end of the simulation.

We concluded that the NuBBE-1970–NSP15 complex was stabler and more robust after binding with NuBBE-1970. Moreover, this work was performed for comparative studies of two potential ligands to find the binding affinity with the protein, and our docking results suggested that NuBBE-1970 binds better. To confirm observation, we performed a molecular-dynamics simulation to check the conformational changes that occur after binding with both complexes. After 15 ns of simulation, a very stable trajectories were achieved, which confirmed that there no more conformational changes occurred during the whole length of simulation.

## 4. Materials and Methods

### 4.1. Data Sources and Preparation

The Nuclei of Bioassays, Ecophysiology and Biosynthesis of Natural Products Database (NuBBE) was accessed, and only phytocompounds (*n* = 1624) were downloaded. The NuBBE database has proven to be a major platform for research in drug design. The content is publicly accessible and comprises validated multidisciplinary information, chemical descriptors, species origins, geographical locations, spectroscopic data, and pharmacological properties [33]. These compounds were downloaded, imported into Discover Studio 2020 in mol2 format, and processed using the ligand-preparation tool.

### 4.2. Target Structure Preparation

The crystal structure of NSP15 endoribonuclease (PDB ID: 6VWW) was retrieved from the protein data bank (https://supplementary.rcsb.org/structure/6VWW) and resolved at 2.20 Å [34]. The structure was downloaded in PDB format as homo 6-mer. Water molecules were deleted, and the 3D structure was prepared in monomer form using the Discover Studio (DS) 2020 protein-preparation wizard and saved in pdb format for further screening purposes.

### 4.3. Virtual Screening and Docking Experiment

From the multistep route of virtual screening of the prepared NuBBE library, we filtered out the 10 best hits via successive screening by LibDock, docking optimization by CDOCKER, and final scoring by calculating binding energies using inbuilt tools in DS 2020. The GOLD program was also used to screen the prepared NuBBE library to explore the full range of ligand conformational flexibility with partial flexibility of the protein [35,36].

Indepth interaction analysis of the top-ranked molecules with the active site of NSP 15 was performed using Auto Dock Tools [37]. The grid was set using x, y, z of 60, 60, 60, and x, y, z centers −69.377, 26.349, and −34.546, respectively. For docking, the final ligand and protein were prepared using Auto Dock tools. The remaining parameters of the program were kept as their defaults considering a movable ligand and rigid protein. The docking result was visualized using DS 2020.

### 4.4. Molecular-Dynamics Simulation

GROMACS 4.6.7 packages [38,39] were used for preparing the system and performing MD simulations using the gromos53a6 force field [40]. The protein solute was solvated by explicit SPC216 water [41] in a dodecahedron box with a margin of 10 Å between solute and box walls. Systems were brought to neutrality by the addition of sodium counter ions. A 1 nm cut-off distance was taken under the particle-mesh Ewald method [42] to calculate long-range electrostatic interactions, and a 1 nm cut-off distance was considered to evaluate van der Waals interactions. The LINCS algorithm of fourth-order expansion was used to constrain bond lengths [43]. The steepest-descent algorithm was applied to optimize for 10,000 steps to remove steric clashes between atoms. The system was equilibrated for 1 ns with position restraints of all heavy atoms. Berendsen weak-coupling schemes were used to maintain the system at 300 K and 1 atom using separate baths for the system. Initial velocities were randomly generated using Maxwell–Boltzmann distribution corresponding to 300 K. Lastly, the production run was performed for 60 ns. Furthermore, xmgrace (http://plasma-gate.weizmann.ac.il) was used for preparing graphs. Ligand-topology preparation was implemented by using the PRODRG server with the option of choosing no chirality, full charge, and no energy minimization [44]. The equilibrated structure obtained after equilibration was considered as the reference structure, and trajectories were fitted to the backbone of this structure. VMD was used to load and visualize all frames of the principal components.

## 5. Conclusions

Due to its growing infection and mortality rates, with no approved drugs or vaccines available, the COVID-19 pandemic is a serious public-health threat. The purpose of this research was to computationally explore natural substances that could potentially inhibit SARS-CoV-2 NSP15, a well-known protein for viral replication in host cells. After multiscoring virtual-screening cascade using DS and GOLD tools, followed by molecular-docking and MD simulations of the Natural Compounds Library (NuBBE database) against NSP15, we described the top-ranked compounds, NuBBE-1970 and NuBBE-242, showing the strongest binding and stable complexation with NSP15. Eleven aa of the NSP15 active site interacted with NuBBE-1970, while 13 aa were found to interact with NuBBE-242. Molecular-dynamics analysis showed that NuBBE-1970 was stabler with NSP-15 than NuBBE-242 was. These compounds were found to be nontoxic and to satisfy drug-likeness properties in our in silico analysis. On the basis of our results, we conclude that these natural compounds should be considered for further studies in the search for COVID-19 therapies.

## Figures and Tables

**Table 1:** Interacting amino acid residues of NSP15 with NuBBE-1970 and NuBBE-242.

Interactions	Target	NuBBE-1970	NuBBE-242
H Bond	NSP15	THR167:HN—UNK0:O32 ASN200:HN—UNK0:O29 ARG207:HE—UNK0:O18	UNK0:H52—PRO206:O UNK0:H55—GLY165:O ARG207:HE—UNK0:O26
Hydrophobic interaction		VAL166—UNK0 UNK0—ILE169 UNK0—ARG199 UNK0—LYS205	ARG199:HD1—UNK0:O26 LYS205:HE2—UNK0:O25 THR167:HN—UNK0 UNK0—VAL166
Electrostatic interaction		GLU203:OE1—UNK0.	—
Total amino acid residues		GLY165, VAL166, THR167, LEU168, ILE169, ARG199, ASN200, GLU203, PHE204, LYS205, and ARG207,	LYS90, GLY165, VAL166, THR167, LEU168, ILE169, GLN197, SER198, ARG199, ASN200, LYS205, PRO206, and ARG207

**Table 2:** Result of molecular docking of NuBBE-1970 and NuBBE-242 with active site of NSP15 by Autodock.

Compounds Name	Binding Energy (kcal/mol)	Inhibition Constant (µM)	Intermolecular Energy (kcal/mol)	Van der Waals, Hydrogen Bond, and Desolvation Energy(kcal/mol)	Electrostatic Energy (kcal/mol)
NuBBE-1970	−6.28	24.98	−8.52	−8.30	−0.22
NuBBE-242	−5.72	64.48	−8.49	−8.45	−0.02

**Table 3:** Pharmacokinetic properties of NuBBE-1970 and NuBBE-242.

Properties		NuBBE_242	NuBBE_1970
Physicochemical	Formula	C22H18O11	C28H22O7
	Molecular weight	458.37 g/mol	470.47 g/mol
	Number of heavy atoms	33	35
	Number of aromatic heavy atoms	18	24
	Number of rotatable bonds	4	4
	Number of H-bond acceptors	11	7
	Number of H-bond donors	8	6
	Molar refractivity	112.06	132.27
	TPSA	197.37 Å^2^	130.61 Å^2^
Lipophilicity (Log Po/w)	iLOGP	1.83	2.40
	XLOGP3	1.17	5.05
	WLOGP	1.91	4.81
	MLOGP	−0.18	2.33
	SILICOS-IT	0.57	3.98
	Consensus Log Po/w	1.06	3.71
Water solubility	Log S (ESOL)	−3.56 (soluble)	−6.18 (poorly soluble)
	Log S (Ali)	−4.91 (moderately soluble)	−7.53 (poorly soluble)
	Log S (SILICOS-IT)	−2.50 (Soluble)	−6.27 (poorly soluble)
Pharmacokinetics	Log Kp (skin permeation)	−8.27 cm/s	−5.58 cm/s
	GI absorption	Low	Low
	BBB permeant	No	No
	CYP2C9 inhibitor	No	Yes
	AMES toxicity	No	No
	hERG I inhibitor	No	No
	Hepatotoxicity	No	No
	Skin sensitization	No	No
	Minnow toxicity	5.305 (log mM)	1.927 (log mM)
Druglikeness	Lipinski	2 violations: NorO > 10, NHorOH > 5	1 violation: NHorOH > 5
	Ghose	Yes	1 violation: MR > 130
	Veber	1 violation: TPSA > 140	Yes
	Egan	1 violation: TPSA > 131.6	Yes
	Muegge	3 violations: TPSA > 150, H-acc > 10, H-don > 5	2 violations: XLOGP3 > 5, H-don > 5
	Bioavailability score	0.17	0.55
Medicinal chemistry	PAINS	1 alert	0 alert
	Brenk	1 alert	1 alert
	Leadlikeness	1 violation: MW > 350	2 violations: MW > 350, XLOGP3 > 3.5
	Synthetic accessibility	4.20	4.48

**Table 4:** Top ten screened compounds based on binding-energy estimation by DS 2020.

Compound	Binding Energy	Ligand Energy	Protein Energy	Complex Energy	Complex Entropy	Protein Entropy	Ligand Entropy	Entropic Energy	C Docker Energy	C Docker Interaction Energy
NuBBE_242	−483.68	162.69	−14,112.7	−14,433.7	−31.45	−31.43	−20.34	20.33	45.56	57.05
NuBBE_1970	−305.87	78.97	−14,088.5	−14,315.4	−31.45	−31.43	−20.81	20.80	22.42	55.35
NuBBE_1485	−296.971	122.49	−14,120.4	−14,294.9	−31.45	−31.43	−20.06	20.05	8.61	51.05
NuBBE_1966	−295.89	97.28	−14,122.8	−14,321.4	−31.45	−31.43	−20.77	20.76	31.35	58.23
NuBBE_1078	−287.46	77.71	−14,129.4	−14,339.1	−31.44	−31.43	−19.47	19.46	6.98	37.12
NuBBE_1969	−280.30	99.14	−14,130.7	−14,311.9	−31.45	−31.43	−20.43	20.41	−10.80	44.28
NuBBE_1263	−277.71	29.814	−14,110.4	−14,358.3	−31.44	−31.43	−19.77	19.76	22.55	40.49
NuBBE_1969	−277.03	95.89	−14,122.6	−14,303.7	−31.45	−31.43	−20.48	20.46	−6.83	45.60
NuBBE_1770	−270.24	35.044	−14,080	−14,315.2	−31.44	−31.43	−19.44	19.43	−4.63	35.99
NuBBE_2205	−268.32	230.91	−14,113.1	−14,150.5	−31.45	−31.43	−20.23	20.22	−94.30	59.87

## Supplementary Materials

The following are available online, Video S1: 1.mp4 and 2.mp4.
