# Supplementary material for: Identification of Persuasive Antiviral Natural Compounds for COVID-19 by Targeting Endoribonuclease NSP15: A Structural-Bioinformatics Approach
Source: Molecules. 2020 Dec 1;25(23):5657. doi: 10.3390/molecules25235657 (PMC7729992; doi:10.3390/molecules25235657)
Supplement: Supplementary file 1 [file molecules-25-05657-s001.zip › English-Editing-Certificate-22783.pdf]

We certify that the following article

Identification of persuasive anti-viral natural compounds for COVID-19 by targeting endoribonuclease NSP15: A structural bioinformatics approach

Mohd Saeed

has undergone English language editing by MDPI. The text has been checked for correct use of grammar and common technical terms, and edited to a level suitable for reporting research in a scholarly journal.
MDPI uses experienced, native English speaking editors. Full details of the editing service can be found at
► https://www.mdpi.com/authors/english.

MDPI Multidisciplinary Digital Publishing Institute

Basel, Switzerland
September 2020

Dr. Shu-Kun Lin
Publisher & President
MDPI
